# Discomfort, pain and stiffness: what do these terms mean to patients? A cross-sectional survey with lexical and qualitative analyses

**DOI:** 10.1186/s12891-022-05214-y

**Published:** 2022-03-24

**Authors:** Martha Funabashi, Simon Wang, Alexander D Lee, Felipe C. K. Duarte, Brian Budgell, Peter Stilwell, Sheilah Hogg-Johnson

**Affiliations:** 1 Canadian Memorial Chiropractic College, 6100 Leslie St., Toronto, ON M2H 3J1 Canada; 2 Université du Québec À Trois-Rivières, 3351 boulevard des Forges, Trois-Rivières, QC Canada; 3 McGill University, 845 Sherbrooke St W, Montreal, QC Canada; 4 University of Toronto, 27 King’s College Circle, Toronto, ON Canada; 5 Ontario Tech University, 2000 Simcoe Street North, Oshawa, ON Canada

**Keywords:** Pain, Discomfort, Stiffness, Patient perspective, Chiropractic

## Abstract

**Background:**

While pain is often the focus of clinical interventions, other clinical outcomes (e.g., discomfort, stiffness) might also contribute to patients’ functionality and well-being. Although researchers and clinicians may view discomfort, pain and stiffness as different constructs, it remains unclear how patients perceive and differentiate between these constructs. Therefore, the purpose of this study was to explore patients’ perceptions of pain, discomfort, and stiffness.

**Methods:**

Chiropractic patients were invited to complete an online cross-sectional survey and describe what ‘discomfort’, ‘pain’ and ‘stiffness’ meant to them using their own words. Lexical and inductive qualitative content analyses were conducted independently and then triangulated.

**Results:**

Fifty-three chiropractic patients (47.2% female, mean age: 39.1 ± 15.1 years) responded. The most common combinations of words to describe discomfort were “can be ignored” and “less severe than”. “Cannot be ignored” and “sharp shooting” were used to describe pain. “Limited range of motion” was used to describe stiffness. Qualitatively, five themes were developed: impact, character, feeling, intensity and temporality. Stiffness was described as limited movement/mobility. Although discomfort and stiffness impacted patients’ activities, patients remained functional; pain was described as stopping/limiting activities. Discomfort was described as dull and tingling, pain as sharp and shooting, and stiffness as tight and restricted. Patients felt displeased and annoyed when experiencing discomfort and stiffness but hurt and in danger of harm when experiencing pain. Discomfort and stiffness were described as less intense than pain, with shorter/intermittent duration; however, all constructs could be experienced constantly.

**Conclusion:**

Patients perceived discomfort, pain and stiffness as different, yet overlapping constructs. This preliminary work advances our knowledge of how patients conceptualize these constructs, contributing to better understanding of what patients mean when reporting these experiences, potentially improving the clinician-patient communication.

**Supplementary Information:**

The online version contains supplementary material available at 10.1186/s12891-022-05214-y.

## Introduction

Health care professionals, including chiropractors, provide clinical care to patients, aiming to improve pain or other relevant clinical outcomes in order to enable patients’ achievement of functional activities. While pain has been the main clinical outcome investigated to date, other clinical outcomes, such as discomfort and stiffness, might also contribute to patients’ functionality, making them equally important. Clearly delineating these constructs may add precision to patient assessment, enhance clinician-patient communication, and enable more targeted and personalized treatment and outcome measurement.

Although pain is often associated with discomfort, it seems that discomfort can be experienced independent of pain. Other non-pain-related experiences that are subjectively unpleasant, such as fatigue, are commonly associated with discomfort [1–3]. Despite being a common experience, the concept of discomfort has not been clearly defined in the literature [1]. A previous study conducted a concept analysis to theoretically clarify the concept of discomfort and delineate the difference between discomfort and other concepts (such as pain). As a result, these authors defined discomfort as “a negative physical and/or emotional state, causing unpleasant feelings or sensations” [1].

Pain and discomfort, however, are not always differentiated. In fact, previous investigations did not make a distinction between these two constructs, and studies either measured discomfort together with pain [4–6], used pain and discomfort as synonyms [7], or assessed both constructs within the same continuum [8, 9]. The concept of pain has been well investigated and a number of definitions exist. According to the newly proposed definition of pain from the International Association for the Study of Pain, it is defined as: “an unpleasant sensory and emotional experience associated with, or resembling that associated with, actual or potential tissue damage” [10]. While definitions of discomfort and pain are somewhat similar, reference to tissue damage is a differentiator between the two concepts [1].

In addition to discomfort and pain, stiffness is another experience commonly reported by patients with musculoskeletal conditions. As with discomfort, previous investigations have not differentiated between stiffness and pain, and have measured these two constructs together [11]. Stiffness is a challenging construct as it has been conceptualized in two ways: 1) as a subjective experience (“feeling” stiffness), and 2) as a physical state that can be objectively measured (“having” stiffness) [12]. It remains unclear how patients conceptualize their subjective experience of stiffness in relation to other constructs, whereas objective stiffness is better understood and is broadly defined as the resistance of an object or a body to a change in length [13]. Previous studies have reported the association between pain and both objective and subjective stiffness [14]. Interestingly, subjective self-perceived back stiffness is poorly related to objective measures of back stiffness and, as with pain and discomfort, the perception of stiffness is multisensorial [12, 15]. The severity and life-impact of subjective stiffness have been quantified using structured questionnaires (e.g., Musculoskeletal Health Questionnaire and Musculoskeletal Stiffness Questionnaire) [11, 14]. However, much work is needed to better understand the descriptors and characteristics of this construct from the patient’s perspective.

While clinicians may view discomfort, pain and stiffness as different constructs, it is not known if patients share the same view. Different understandings can lead to miscommunication and misunderstandings in the clinical encounter, where clinicians and patients interpret different experiences based on their own definitions. By elucidating patients’ perceptions towards discomfort, pain and stiffness constructs, clinicians would better understand what patients actually mean when using these concepts, enhancing the clinician-patient communication. Consequently, more accurate interpretation and utilization of such patient reported experiences will be possible, potentially advancing the quality of care.

Therefore, the purpose of this study was to explore patients’ perceptions of discomfort, pain and stiffness concepts and if/how patients differentiate between them.

## Methods

This study used a cross-sectional online survey and was approved by the Canadian Memorial Chiropractic College (CMCC) Research Ethics Board (#2006X03) and performed in accordance with the ethical standards as laid down in the 1964 Declaration of Helsinki and its later amendments or comparable ethical standards.

A convenience sample of adult patients (≥ 18 years old) receiving care at three private chiropractic clinics in the Toronto area and at CMCC’s campus clinic were invited to participate by their clinician. If interested, the patient was introduced to the principal investigator who provided detailed information about the study. If the patient agreed to participate, the survey link was sent via email.

The survey was specifically developed for this study and was available electronically between 4^th^ November and 23^rd^ December 2020 via the SurveyMonkey electronic data capture platform (Momentive Inc., San Mateo, California, USA; www.surveymonkey.com). The opening page of the survey provided information regarding the purpose and content of the study, potential risks and benefits of participation, expected duration to complete the survey, voluntary participation, confidentiality and the research team’s contact information. All participants provided their informed consent electronically after reading the study information and prior to participating in the study.

The survey included three main sections (supplementary file 1). The first section collected information on whether the patient was experiencing each of the constructs (discomfort, pain and stiffness) at the time they were responding to the survey [Yes/No for each construct], location of these experiences [checkboxes of body areas, able to check all that apply] and their intensity [electronic visual analogue scale (VAS), where 0 corresponded to ‘most comfortable/no pain/no stiffness’ and 100 corresponded to ‘most uncomfortable/worst pain/worst stiffness’]. The second section asked participants to use their own words to describe what ‘discomfort’, ‘pain’ and ‘stiffness’ meant to them. The third section collected participants’ demographic information (including age, sex, condition they were receiving care for and its duration).

The survey instrument underwent expert review by three practising chiropractors for face- and content-validation. The survey was refined based on the clinicians’ feedback, and the finalised online survey version was estimated to take approximately 5–10 min to complete.

### Data analysis

Quantitative data from sections one and three of this survey were analyzed descriptively. Specifically, counts and percentage of participants who responded “yes” to each of the experiences (discomfort, pain and stiffness) were calculated. Means ± standard deviations (SD) were calculated for demographic characteristics as well as for each experience’s VAS score. Counts and percentage of participants reporting each experience in each body part were also calculated.

The open-ended responses concerning each construct (discomfort, pain and stiffness) were analyzed independently with a lexical and a qualitative approach. For the lexical analysis, the 3 corpora (aggregated sets of entries describing each construct: discomfort, pain and stiffness) were first analyzed separately using the online resource Vocabprofile (https://www.lextutor.ca/vp/eng/) [16], to identify the frequency of each unique word (termed a ‘type’ by linguists). To clarify this terminology, in the sentence “the doctor treated the patient,” there are 5 ‘tokens’, i.e. separate words in the text, but only 4 types, because one of the types—‘the’ – occurs twice. Each type was classified according to whether it was from among the General Service List (GSL) – the approximately 2,000 most common word families in the English language [17], from among the Academic Word List (AWL) – the approximately 570 word families common in academic settings [18], or off-list – not from among either the GSL or AWL. Off-list words are often technical words or hold special meaning within their source corpus [19]. Analysis for ‘lexical closure,’ by plotting types versus tokens [20], confirmed that the corpora were too small to be accepted as representative of patient language in general. Lexical closure is the point at which adding more words (tokens) to the corpus (collection of text) does not result in the addition of any new words (types). Expressed another way, the sample of language contains all of the unique words (types) that would be found in the entire language that the sample is intended to represent.

**Fig. 1:**
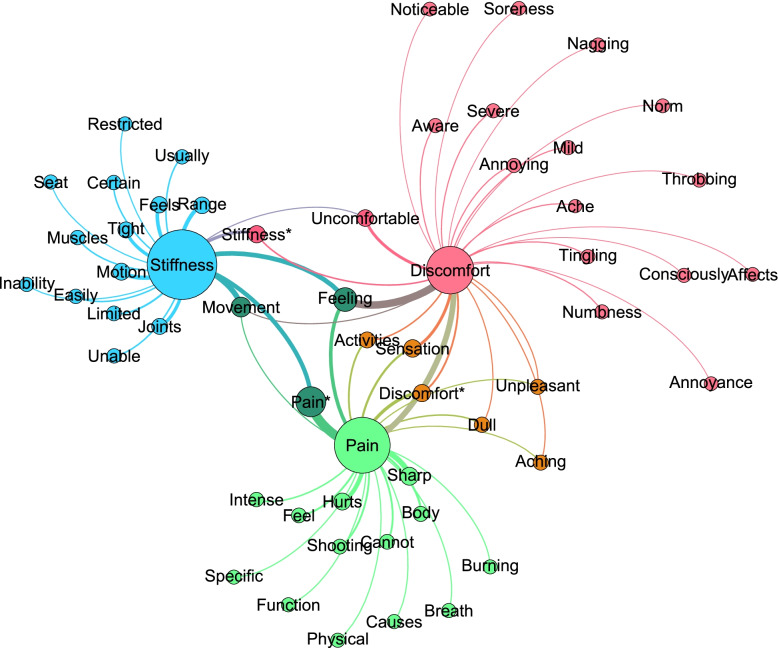
Mapping of keywords of each construct

The 3 corpora were analyzed using the WordSmith Tools 8.0 WordList (WordSmith Tools version 8. Lexical Analysis Software: Stroud; 2020) utility versus the ‘Basic English’ stoplist to produce lists of ‘meaningful words’. Word lists were then analyzed with the KeyWords utility versus the New York Times sub-corpus of the American National Corpus to produce a list of Keywords – words which were statistically over-represented in each corpus (discomfort, pain, stiffness). Additionally, the cluster function in WordSmith Tools was used to identify N-grams, frequently repeated 2- 3- and 4-word phrases. Subsequently, each keyword or phrase was plotted in the social networking program Gephi (https://gephi.org/) [21], with the subdomains (discomfort, pain, stiffness) as sources and the keywords and phrases as targets (Fig. 1).

For the qualitative analysis, an inductive (conventional) content analysis approach [22] was used to categorize patients’ perceptions of each construct. Two investigators (MF, SW) conducted independent analyses by individually coding each open-ended response. The two investigators individually coded batches of 10–20 responses, met via videoconference to review coding, refine thematic categories, and reach coding consensus. An expert in qualitative research (PS) provided coding guidance, helped resolve coding discrepancies, and provided input during category (theme) development. Representative quotes with identifiers illustrate participants’ perceptions of each construct. Microsoft Excel spreadsheets (Microsoft Corporation, USA) were used for individual coding and a master shared online spreadsheet was maintained with combined coding decisions.

## Results

### Participant characterization

The survey link was sent to 55 patients, and a total of 53 patients responded to the survey. Characteristics of patients who participated in this study are presented in Table 1. Most patients were receiving chiropractic care for low-back (54.7%), neck (47.2%) and shoulder (41.5%) complaints, and experiencing discomfort, pain or stiffness for over 3 months (71.7%).

**Table 1:** Characteristics of participating patients (*n* = 53)

**Characteristic**	
Female (n, %)	25 (47.2%)
Age (years, mean ± SD)	39.1 ± 15.1
**Receiving chiropractic care for (n, %):**	
Head	4 (7.5%)
Neck	25 (47.2%)
Shoulder	22 (41.5%)
Upper extremity	8 (15.1%)
Mid-back	18 (34%)
Low back	29 (54.7%)
Hip / pelvis	14 (26.4%)
Lower extremity	16 (30.1%)
**Duration of condition/complaint (n, %)**	
< 3 months	13 (24.5%)
> 3 months	38 (71.7%)

Table 2 presents the counts and percentages of participants reporting each experience (discomfort, pain and stiffness) and their respective locations. The most common location in which patients reported experiencing discomfort, pain and stiffness were the low back and lower extremity, with discomfort and stiffness also being commonly reported in the neck.

**Table 2:** Counts and percentages of participants experiencing each symptom by location (*n* = 53)

	**Discomfort**	**Pain**	**Stiffness**
Experiencing [Yes]	43 (81.1%)	28 (52.8%)	37 (69.8%)
**Location (n, %)**^a^			
Head	5 (11.6%)	3 (10.7%)	1 (2.7%)
Neck	22 (51.2%)	6 (21.4%)	14 (37.8%)
Shoulder	20 (46.5%)	5 (17.9%)	12 (32.4%)
Upper extremity	11 (25.6%)	3 (10.7%)	3 (8.1%)
Mid-back	11 (25.6%)	5 (17.9%)	12 (32.4%)
Low back	27 (62.8%)	11 (39.3%)	21 (56.8%)
Abdomen	1 (2.3%)	0	1 (2.7%)
Hip / pelvis	13 (30.2%)	8 (28.6%)	8 (21.6%)
Lower extremity	23 (53.4%)	17 (60.7%)	13 (35.1%)
**VAS (mean ± SD)**	38.4 (± 19)	40.8 (± 21.2)	42.8 (± 22.2)

*VAS* visual analogue scale

^a^percentage based on the number who indicated experiencing the construct (‘Experiencing [Yes]’)

### Lexical analysis

The word counts for each corpus (tokens) were well below the sizes required for meaningful statistical comparisons [23]. Other descriptive statistics in Table 3 suggest that the actual contents (patients’ entries) were more characteristic of ‘general’ versus ‘technical’ English [19].

**Table 3:** Lexical description of the corpora used to describe each symptom

	**Discomfort**	**Pain**	**Stiffness**
Tokens (word count)	574	771	776
Types (unique words)	229	319	291
Type/Token Ratio	0.04	0.41	0.38
Token/Type Ratio	2.51	2.42	2.67
Lexical Density	0.51	0.55	0.56
GSL (%)	86.4	83.79	88.14
AWL (%)	5.75	7.91	5.80
Off-list (%)	7.84	8.30	6.06

GSL (%) = percentage of tokens from among the General Service List, AWL (%) = percentage of tokens from among the Academic Word List, Off-list (%) = percentage of words from neither the GSL or AWL

Lexical closure analysis [20] revealed that for each construct, corpus size was insufficient to be taken as representative of patient language in general. From the keywords and N-gram lexical analysis, the most common combination of words to describe discomfort were “can be ignored”, “less severe than” and “feeling of/that”. “Cannot be ignored” and “sharp shooting” were most commonly used to describe pain. “Limited range of motion” was commonly used to describe stiffness.

Figure 1 presents the mapping of keywords onto each construct, where node size is scaled to prevalence of each word (type) in the combined corpora and edges (curved lines) indicate which constructs map onto which keywords. Edge thickness is scaled to the number of times each keyword occurs within each of the corpora. In the small corpora used in this study, most keywords were associated with only one construct, although the keywords ‘movement’ and ‘feeling’ were associated with all three constructs.

### Qualitative analysis

**Fig. 2:**
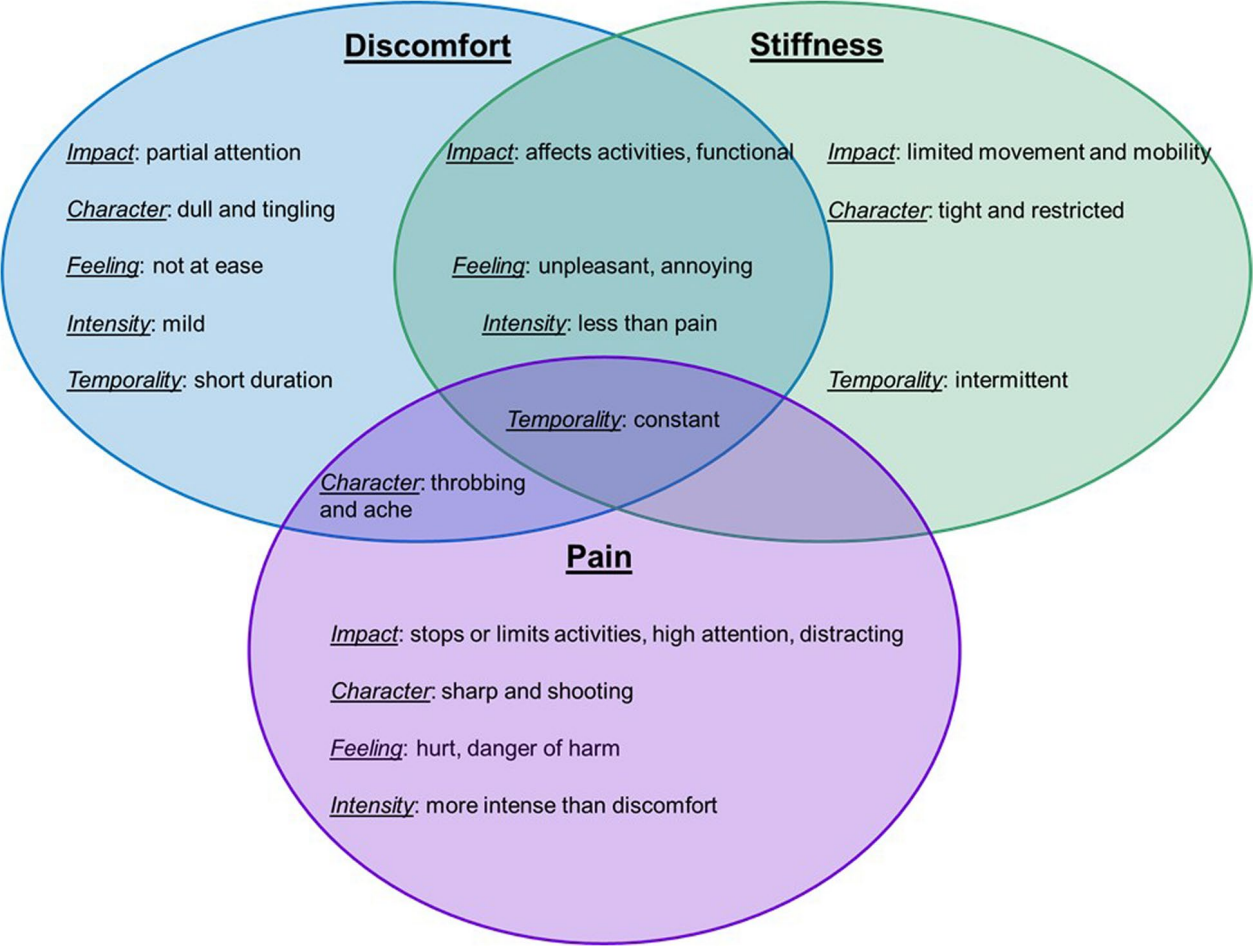
Overview of the relational aspect of major qualitative themes for each construct

Coding of participants’ responses resulted in the identification of 5 major themes to describe each construct: impact, character, feeling, intensity and temporality. Figure 2 provides an overview of the relational aspect of the major themes for each construct, their differentiation and the overlap between them.

#### Impact

The impact theme described how discomfort, pain and stiffness impacted activities. Specifically, patients reported that, when they experienced discomfort, it required only partial attention and that they were consciously aware of it: *“When you are consciously aware of your body and know its not functioning optimally. You are aware of the issue”* [021]. One participant compared discomfort and pain and explained that, while discomfort can be ignored: *“A sensation out of the ordinary that can be ignored”* [025], pain cannot: *“A negative sensation that cannot be ignored”* [025]. Another participant agreed: *“Pain is like a headache in any part of your body. It hurts and is constantly on your mind. […] When pain is occurring, it is always at the forefront of my mind”* [040].

There was an overlap between discomfort and stiffness and while both were described as affecting participants’ activities, they allowed participants to remain functional: *“Can function during activities of daily living but it doesn’t feel ‘normal’”* [012]. Pain, on the other hand, was described as stopping or limiting their activities: *“stops you performing the activities you need to do”* [009]. Most participants also described stiffness as limiting their movement and mobility: *“When the movement in certain parts of the body are restricted”* [033].

#### Character

Participants recognized that each construct had a particular character and specific adjectives were commonly used to describe each construct. Discomfort was described as having a dull and tingling character *(“tingling, numbness”* [011]), pain was described as sharp and shooting (“*Sharp, distracting, distressing”* [031]), and stiffness was described as tight and restricted (*“tight, limited range of motion, stuffy feeling within”* [030]). One participant compared all three experiences in terms of their character: *“Discomfort to me is almost a dull ache. Not something that is debilitating but something that is noticeable and is not usual. Pain to me is when there are sharp shooting sensations or feelings of discomfort that become unbearable. Stiffness to me is when something is a bit tight or hasn’t been stretched properly. When I move a certain way and there is resistance but no pain”* [044]. Interestingly, there was an overlap between discomfort and pain: both were described as having a throbbing and aching character, whereas discomfort was *“Mild ache”* [049] and pain was *“aching, pinching, throbbing, stabbing feeling”* [004].

#### Feeling

Participants reflected on the emotional experience of how each construct made them feel. Most participants described they felt not at ease when experiencing discomfort: *“A general unease, a feeling of unsettledness”* [032]. They also described discomfort and stiffness as unpleasant and annoying. For instance, one participant reported that discomfort was *“When I’m unable to find a position that relieves the sensation, an uncomfortable feeling that is more so annoying than painful”* and stiffness was *“Usually in a joint region, maybe a decrease in ROM* [range of motion]*, annoying during everyday activities but doesn’t cause me to stop doing them”* [024]. On the other hand, participants indicated that pain made them feel hurt *“Feeling of hurt”* [037] and in danger of harm: *“[…] I feel like pain often goes deeper than discomfort and to me signals damage or something wrong with the area. To me when I think of pain it is associated with an injury or harmful stimulus”* [027].

#### Intensity

Participants often compared and differentiated between discomfort, pain and stiffness in terms of intensity. Discomfort and stiffness were both described as less intense than pain, with discomfort being *“hurting but less severe than pain, can be ignored or distracted from fairly easily”* [042] and stiffness being *“A symptom less serious than pain that, although uncomfortable, generally does not restrict your ability to carry out activities of daily living”* [016]. However, one participant described stiffness being *“Between discomfort and pain”* [036]. Pain was described as more intense: “*Hurt, intense*” [047]. One participant compared discomfort and pain, and described discomfort as an *“adjective, less severe than pain”* and pain as the *“strongest adjective to describe aches, soreness”* [039].

#### Temporality

Participants also considered the temporality of each construct. All constructs were described as potentially constant, with discomfort being *“on-going, annoying reminders”* [043], pain being a *“Constant ache sensation”* [017], and stiffness being *“Feeling uncomfortable constantly, make me move slowly”* [038]. However, some participants perceived discomfort to be short in duration: *“Discomfort to me is somewhere beneath pain. Its an uncomfortable or unpleasant sensation, often very short in length. Discomfort to me is usually brought on by a stimulus, like a pair of pants that are too tight”* [027]. Stiffness was described as both constant and intermittent: *“Constant as well as intermittent achiness”* [049].

## Discussion

With a cross-sectional survey, this study explored chiropractic patients’ perceptions of three constructs commonly reported in clinical practice: discomfort, pain and stiffness. Lexical and qualitative analyses revealed that patients perceived discomfort, pain and stiffness as somewhat different constructs, although overlap was identified. Specifically, discomfort and stiffness were described as impacting patients’ activities, however, they remained functional; whereas pain was described as stopping/limiting activities. Patients described feeling displeased and annoyed when experiencing discomfort and stiffness, but hurt and in danger of harm when experiencing pain. Discomfort and stiffness were described as less intense than pain, with shorter/intermittent duration; however, all constructs could be experienced constantly. This study contributes to better understanding what patients actually mean when using these concepts in the clinical encounter.

The techniques employed in this study demonstrate that it is practical to conduct a lexical analysis of patient free-text entries, and to characterize the lexicons used by patients to describe different clinical constructs. In the small corpora derived in this study, patients appeared to use ‘general’ rather than ‘technical’ language to describe their experiences, and used lexicons which were largely specific to the different constructs under investigation. Thus, while our own corpora were too small to justify statistical comparisons, the trends that we saw toward lexical closure suggest that with samples from hundreds, not thousands or millions, of patients, we would have corpora which were convincingly representative of the broader language. Therefore, lexical analysis of adequately sized corpora appears practical, and the results could assist in facilitating patient-clinician communications, including history-taking and formulation of diagnoses.

Although lexical and qualitative analyses were conducted independently to avoid cross-contamination, results from the lexical analysis provided quantitative support to themes developed from the qualitative analysis. Specifically, while the qualitative analysis provided a more in-depth description of patients’ experience related to discomfort, pain and stiffness, lexical analysis provided quantitative support, emphasizing the Intensity, Character and Impact themes with the words patients chose to use to respond to our survey.

There is limited evidence regarding the concept and perceptions of discomfort, especially in musculoskeletal conditions, which limits our ability to compare our results with the literature. This lack of research related to discomfort is likely due to the investigative focus on pain. In fact, our results show that patients usually remain functional when experiencing discomfort. Given that most rehabilitative research is focused on bringing the patient to a functional state, relief of discomfort might not be perceived as an important outcome in rehabilitation. However, a high level of musculoskeletal discomfort has been reported to be a predictor of future musculoskeletal pain in workers [24], highlighting the importance of discomfort as a clinical outcome that should get more attention. Additionally, moving away from a problem-based and towards a quality improvement mindset, improving processes and interventions to enhance all possible clinical outcomes (including discomfort) may contribute to enhancing quality of care and patient satisfaction.

Interestingly, self-report questionnaires commonly used in clinical investigations, such as the McGill Pain Questionnaire and Musculoskeletal Health Questionnaire, do not clearly differentiate between discomfort, pain and stiffness. Our results indicate that patients make specific distinctions between these constructs, which might not be captured by these questionnaires. This highlights a limitation of currently available questionnaires and revisions could potentially be implemented to better align questionnaires with patients’ nuanced understandings of these constructs, enhancing clinical assessment and interpretation of patients’ responses.

Our results suggested that patients perceived that pain was an indicator of potential injury or harm; however, some patients also described pain as the feeling of being hurt, which can be interpreted in a non-physical context. It is well known that pain is multifactorial and that it cannot be solely inferred from the state of bodily tissues [25–28]. Indeed, both the revised definition of pain from the International Association for the Study of Pain and other recent evidence suggest that although pain is often associated with physical harm, pain is also influenced by cognitive, emotional, psychological and social factors [10, 25, 26]. Consequently, pain is a highly personal experience making it very challenging to conceptualize and measure [26]. The literature also defines discomfort and pain more generally, often including physical, psychological and emotional aspects [1, 10]. Noticeably, the responses in this study focused on the physical aspect of these two concepts and are consistent with previous studies investigating patients’ perceptions of pain, reporting that they often talk about pain as a sign of a “physical” issue or bodily dysfunction [29–31]. Our results contribute to better understanding how patients perceive pain, specifically, and provide additional knowledge on how they differentiate pain from other unpleasant experiences, such as discomfort and stiffness.

Results from this study indicate that participating patients perceived the concept of stiffness to be closely related to reduced or restricted movement, mobility and range of motion. This is in accordance with previous findings reporting that stiffness was described as a perceived resistance to movement and a lack of movement velocity [12, 32]. Stanton et al. [12] suggested that the conscious perception of stiffness may represent a multisensory perceptual inference and is not derived exclusively from joint relevant sensory information. Indeed, previous studies showed that subjective or self-reported stiffness did not correlate with objective measures of stiffness [12, 15]. This indicates that, while a unique concept related to movement restriction, perceived stiffness is likely multifactorial, which may explain the overlap it presented with pain and discomfort concepts.

### Limitations

Lexical closure, the linguistic equivalent of a power analysis, revealed that the corpora of free-text entries used in this exploratory study were much too small to permit meaningful statistical comparisons among the 3 constructs. Our study used a convenience sample, where clinicians’ selection bias is possible. Therefore, our results are specific to our sample and might not represent the perceptions of the general patient population. Our qualitative data contained enough information to develop thematic categories and fulfill our exploratory study aim [33]. However, this is preliminary work focused on patients, and our findings suggest that future studies should be undertaken, including exploring clinicians’ perceptions to complement that of patients. Specifically, qualitative interviews allow for more in-depth responses which in turn, allow for a more detailed analysis that may help us better understand the meanings of these constructs, their specific characteristics (e.g., intensity, duration, etc.), how they feel, as well as other constructs, such as suffering, soreness and ache. These could also inform the development and refinement of existing patient-reported outcome measures and questionnaires that measure discomfort, pain and stiffness for clinical and research purposes. In total, 53 unique responses, consisting of short phrases of few words to full paragraphs of 100 + words in length, were analyzed. We cannot be certain of data adequacy, which limits the extrapolation of our results beyond study participants. Most participants reported experiencing more than one construct at the time they responded to the survey. Although this might portray real-world clinical patient presentation, experiencing more than one construct simultaneously might have contributed to the overlap in qualitative themes observed in our study. Additionally, most patients were seeking chiropractic care for a chronic condition. This can potentially influence their perception of how discomfort, pain and stiffness are different and future studies should explore if patients with acute conditions have a unique perception of these concepts in comparison to chronic patients. Finally, distinct pathophysiological mechanisms of pain (neuropathic, nociceptive or nociplastic) are thought to influence pain profiles in terms of pain quality, spatial characteristics, and pain symptoms. Therefore, pain mechanisms may have also influenced the pain perception in our study.

## Conclusion

Findings from this study suggest that although patients perceive discomfort, pain and stiffness as different constructs, there is some overlap. This preliminary work contributes to better understanding of how patients conceptualize these constructs. By advancing our knowledge regarding what patients actually mean when reporting these experiences in the clinical encounter, a more accurate interpretation and utilization of such reported experiences is facilitated, which may improve clinician-patient communication.

## Supplementary Information

**Additional file 1.** Complete survey that was used in this study.

## Data Availability

The datasets used and/or analysed during the current study are available from the corresponding author on reasonable request.

## Abbreviations

AWL: Academic word list
CMCC: Canadian Memorial Chiropractic College
GSL: General service list
ROM: Range of motion
SD: Standard deviation
VAS: Visual analogue scale
