# Supplementary material for: Discomfort, pain and stiffness: what do these terms mean to patients? A cross-sectional survey with lexical and qualitative analyses
Source: BMC Musculoskelet Disord. 2022 Mar 24;23:283. doi: 10.1186/s12891-022-05214-y (PMC8944041; doi:10.1186/s12891-022-05214-y)
Supplement: Supplementary file 1 — Additional file 1. Complete survey that was used in this study. [file 12891_2022_5214_MOESM1_ESM.pdf]

Comfort, pain and stiffness - a survey on patient perspectives

Information Letter

Study Title: **Comfort, pain and stiffness - a survey on patient perspectives**

Research Team:
Dr. Martha Funabashi
(416) 482-2340 ext. 714
mfunabashi@cmcc.ca

Dr. Simon Wang
Dr. Felipe Duarte
Dr. Alex Lee
Dr. Sheilah Hogg-Johnson

Purpose of the Study: **To explore patients' perceptions of discomfort, pain and stiffness.**

Procedures Involved in this Study and Time Commitment: **You will be asked to respond a 12-question survey about discomfort, pain and stiffness. This survey should take about 5 minutes to complete.**

Potential Risks and Associated Safeguards: **No risks or discomforts related to this study are expected. <u>You are free to answer or not answer any questions.</u>**

Changing Your Mind about Participation: **You may withdraw from the study at any time without any consequences. To do so, simply submit the survey with the responses you agree to provide or do not submit the survey.**

Potential Benefits of Participation: **Information from this survey will help researchers better understand how patients perceive these symptoms. You may withdraw from participation in the study at any time without any consequences.**

Confidentiality: **The survey is completely anonymous and no data that would allow your identification (e.g., name) will be collected. Only the investigators involved in this study will have access to this anonymized data. All data will be stored on computer hard drives (password protected) and/or digital storage media (locked in the investigator's filing cabinet).**

Concerns about Participation: **We would like to assure you that this study has been reviewed by, and received ethics clearance through, CMCC's Research Ethics Board (REB). However, the final decision about participation is yours. In the event you have any comments or concerns resulting from your participation in this study, please contact Mr. Mark Fillery at (416) 482-2340 ext. 267.**

Questions about the Study: **If you have any questions or want more information about this study, please feel free to ask the investigators. If you have additional questions later on, please contact Dr. Martha Funabashi at (416) 482-2340 ext. 714 or by e-mail at mfunabashi@cmcc.ca.**

Thank you very much,

Drs. Martha Funabashi, Simon Wang, Felipe Duarte, Alex Lee and Sheilah Hogg-Johnson

1. Do you consent to take part in our survey?

- ◯ Yes
- ◯ No

Comfort, pain and stiffness - a survey on patient perspectives

Discomfort

* 2. Are you currently experiencing any discomfort?

- ◯ Yes
- ◯ No
- ◯ Prefer not to answer

Comfort, pain and stiffness - a survey on patient perspectives

Discomfort

3. Please check the areas in which you are currently feeling any discomfort:

- ☐ Head
- ☐ Neck
- ☐ Shoulder
- ☐ Arm / elbow
- ☐ Wrist / hand / fingers
- ☐ Mid-back
- ☐ Chest
- ☐ Low back
- ☐ Abdomen
- ☐ Hip / pelvis
- ☐ Leg / knee
- ☐ Ankle / foot / toes
- ☐ Prefer not to answer

4. Please indicate how much discomfort you are experiencing (if more than one site, rate for the site with most discomfort):

0 - Most comfortable 100 - Most uncomfortable

Comfort, pain and stiffness - a survey on patient perspectives

Pain

* 5. Are you currently experiencing any pain?

- ◯ Yes
- ◯ No
- ◯ Prefer not to answer

Comfort, pain and stiffness - a survey on patient perspectives

Pain

6. Please check the areas in which you are currently feeling pain:

- ☐ Head
- ☐ Neck
- ☐ Shoulder
- ☐ Arm / elbow
- ☐ Wrist / hand / fingers
- ☐ Mid-back
- ☐ Chest
- ☐ Low back
- ☐ Abdomen
- ☐ Hip / pelvis
- ☐ Leg / knee
- ☐ Ankle / foot / toes
- ☐ Prefer not to answer

7. Please indicate how much pain (if more than one site, rate for the site with most pain)

0 - No pain 100 - Worst pain

Comfort, pain and stiffness - a survey on patient perspectives

Stiffness

* 8. Are you currently experiencing any stiffness?

- ◯ Yes
- ◯ No
- ◯ Prefer not to answer

Comfort, pain and stiffness - a survey on patient perspectives

Stiffness

9. Please check the areas in which you are currently feeling any <u>stiffness:</u>

- [ ] Head
- [ ] Neck
- [ ] Shoulder
- [ ] Arm / elbow
- [ ] Wrist / hand / fingers
- [ ] Mid-back
- [ ] Chest
- [ ] Low back
- [ ] Abdomen
- [ ] Hip / pelvis
- [ ] Leg / knee
- [ ] Ankle / foot / toes
- [ ] Prefer not to answer

10. Please indicate how much <u>stiffness</u> (if more than one site, rate for the site with most stiffness):

0 - No stiffness — 100 - Worst stiffness

Comfort, pain and stiffness - a survey on patient perspectives

11. Help us better understand the terms “discomfort”, “pain” and “stiffness”.

Using your own words and the space below, please describe what “discomfort”, “pain” and “stiffness” mean to you:

Discomfort:

Pain:

Stiffness:

Comfort, pain and stiffness - a survey on patient perspectives

Demographic Information

12. What is your sex?

- ◯ Female
- ◯ Male
- ◯ Other
- ◯ Prefer not to answer

13. How old are you? (in years)

[ ]

14. You are receiving chiropractic treatment for:

- ☐ Head
- ☐ Neck
- ☐ Shoulder
- ☐ Arm / elbow
- ☐ Wrist / hand / fingers
- ☐ Mid-back
- ☐ Chest
- ☐ Low back
- ☐ Abdomen
- ☐ Hip / pelvis
- ☐ Leg / knee
- ☐ Ankle / foot / toes
- ☐ Prefer not to answer

15. Overall, how long have you been feeling discomfort / pain / stiffness?

- ◯ < 3 months
- ◯ > 3 months

16. How long have you been receiving treatment at CMCC Clinic?

- ◯ < 3 months
- ◯ > 3 months

Comfort, pain and stiffness - a survey on patient perspectives

Exit Page

Thank you for taking part in our survey, "Comfort, pain and stiffness - a survey on patient perspectives". For any inquiries about the survey, you can contact Dr. Martha Funabashi at mfunabashi@cmcc.ca. For questions about your rights as a participant in this study, please contact Mr. Mark Fillery at mfillery@cmcc.ca, or by phone at 416-482-2340 x267.
